# Pharmacoeconomics of sublingual immunotherapy with the 5-grass pollen tablets for seasonal allergic rhinitis

**DOI:** 10.1186/s12948-017-0058-3

**Published:** 2017-03-07

**Authors:** Carlo Lombardi, Valerie Melli, Cristoforo Incorvaia, Erminia Ridolo

**Affiliations:** 1 Allergy and Pneumology Departmental Unit, Fondazione Poliambulanza Hospital, Brescia, Italy; 2 Department of Clinical & Experimental Medicine, University of Parma, Via Gramsci 14, Parma, Italy; 3 Pulmonary Rehabilitation, Centro Specialistico Gaetano Pini/CTO, Milan, Italy

**Keywords:** Allergic rhinitis, Pharmacoeconomics, Cost-effectiveness, Allergen immunotherapy, Sublingual immunotherapy

## Abstract

Allergic rhinitis has a very high burden regarding both direct and indirect costs. This makes essential in the management of AR to reduce the clinical severity of the disease and thus to lessen its costs. This particularly concerns allergen immunotherapy (AIT), that, based on its immunological action on the causes of allergy, extends its benefit also after discontinuation of the treatment. From the pharmacoeconomic point of view, any treatment must be evaluated according to its cost-effectiveness, that is, the ratio between the cost of the intervention and its effect. A favorable cost-benefit ratio for AIT was defined, starting from the first studies in the 1990s on subcutaneous immunotherapy (SCIT) in AR patients, that highlighted a clear advantage on costs over the treatment with symptomatic drugs. Such outcome was confirmed also for sublingual immunotherapy (SLIT), that has also the advantage on SCIT to be free of the cost of the injections. Here we review the available literature on pharmacoeconomic data for SLIT with the 5-grass pollen tablets.

## Background

The steadily increasing prevalence of allergic disorders, including allergic rhinitis (AR), asthma, and atopic dermatitis, with global figures currently corresponding to more than 20% of the general population [1–4] results in a relevant individual and social economic burden. For example, concerning AR, in a retrospective analysis performed in the 2000s using data from a US health plan covering about 15 million patients, the mean total costs per year related to rhinitis were $657 per patient, the primary contributor being outpatient visits [5]. The economic burden includes direct costs, that are related to drug treatment and visits at physician office, and indirect costs, that are associated to reduced/missed work productivity [6]. In the late 1990s the cost for AR in the US were estimated in $4.5 billion for direct and $3.4 billion for indirect costs, respectively, [7] and by 2005 total expenditures to treat AR reached $11.2 billion [8]. In Europe, a study conducted in 2003 found a mean annual cost of €1089 for child/adolescents and €1543 per adults, respectively, with predominance of indirect costs in adults (about 50%) compared with children (6%), in whom however the estimate did not include school absences [9]. A probabilistic cost of illness study in Italy estimated a global economic burden associated with respiratory allergies and their main co-morbidities of €7.33 billion (95% CI: €5.99–€8.82). A percentage of 27.5% was associated with indirect costs and 72.5% with direct costs [10]. A very recent study from UK on 1000 adults patients with seasonal AR demonstrated that limiting the assessment to absenteeism (on average, 4 days/year) a cost of £1.14 billion/year was estimated [11]. Pharmacoeconomics is the scientific discipline that analyzes the value of different drug therapies, serving to guide the optimal allocation of healthcare resource by standardized and scientifically solid methods [12]. From the pharmacoeconomic point of view, any drug treatment must be evaluated according to its cost-effectiveness, the cost referring to the resource expenditure for the intervention, that is usually measured in pecuniary terms [13]. For example, in AR first generation antihistamines may impair mental performances (due to their sedating effects) more than in untreated patients [14] and thus rise indirect cost. By a global therapeutic approach to AR, any preventive strategy that is aimed at reducing the severity of the rhinitis is likely to lessen its costs, and this particularly concerns allergen immunotherapy (AIT).

## Cost effectiveness of allergen immunotherapy

Allergen immunotherapy is aimed at reducing the symptoms of allergy by increasing the tolerance to the administered allergen and modifying the natural history of the allergic disease [15]. The first pharmaco-economic studies were conducted in the 1990s in patients treated with subcutaneous immunotherapy (SCIT). Their results, that are summarized in Table 1, were favorable, showing significant reductions of direct and indirect costs. The cost saving reaches its maximum when SCIT is stopped after the recommended 3 years of treatment and continues to work due to the persistent modification of the immunological response to the specific allergen. This was apparent in a study on patients from Italy with AR and asthma induced by sensitization to *Parietaria* pollen, who underwent 3 years of SCIT by a *Parietaria judaica* extract or with symptomatic drugs [19]. The patient were evaluated before SCIT initiation and then each year for a period of 6 years during the pollen period of *Parietaria* by measuring the nose, eye, and lung symptom scores, also registering by diary cards the drug consumption. The data obtained showed a significant difference in favor of SCIT plus drug treatment vs. only drug treatment. The cost reduction was about 15% at the 2nd year and 48% at the 3rd year, when a high statistical significance was detected. This was then maintained until the 6th year, i.e. 3 years after discontinuing SCIT, when the reduction of cost was 80%, with a net saving corresponding to €623 per year for each patient at the final evaluation.

**Table 1:** Studies on pharmacoeconomics of subcutaneous immunotherapy

Author (year)	Patients	Allergen	Study duration (years)	Results
Buchner (1995) [16]	Adults	Pollen, mites	10	−54% costs for symptomatic treatment
Schadlich (2000) [17]	Adults	Pollen, mites	10	€332-608 saving per patient
Petersen (2005) [18]	Adults	Pollen	4	€203 saving per patient
Ariano (2006) [19]	Adults	Pollen	6	48% money saving at year 4
Omnes (2007) [20]	Adults & children	Pollen, mites	6	€1327 saving per patient for pollen, €393 for mites
Hankin (2008) [21]	Children	Pollen, mites	1.5	€308 6-month saving per patient
Hankin (2010) [22]	Children	Pollen, mites	1.5	−34% total healthcare cost per patient
Wang (2011) [23]	Adults	Pollen, mites	1.5	−41% total healthcare cost per patient

On the other hand, a recent study showed that the cost-saving may also occur early. In fact, a retrospective analysis based on Florida Medicaid claims estimated the mean 18-month health care cost of 4967 patients with newly diagnosed AR who were treated for the first time with SCIT compared with 19,278 control subjects treated only with drugs [24]. In SCIT-treated patients a mean 18-month total health care cost of $6637 was calculated, compared with $10,644 in controls (38% lower in SCIT-treated, P < 0.0001). Significant savings were detected within 3 months from starting SCIT, with no significant difference between the savings observed in SCIT-treated adults and SCIT-treated children ($4397 vs. $3965). The fact that the more recently introduced sublingual immunotherapy (SLIT) is performed by patients at home and thus is free of the cost of injections suggests that the cost-effectiveness of SLIT may be even better than SCIT.

### Studies on sublingual immunotherapy

The first study, that involved one Allergy center in Italy, evaluated the cost effectiveness of SLIT in pediatric patients with respiratory allergy [25]. A group of 135 children with AR and asthma was studied, the data concerned 1-year prior to receive SLIT and 3-year after starting SLIT. The outcome measures were the number of disease exacerbations, visits, and missed nursery or school days, including direct and indirect costs. Forty-six patients had perennial allergy and 89 had seasonal allergy. All outcome measures showed a considerable reduction during SLIT compared to the previous 1-year period. The annual cost/patient averaged to €2672 before starting SLIT and to €629/year during SLIT, with comparable results for allergen subgroups. These findings suggested that SLIT was able to reduce the global cost of AR. Such outcome was confirmed in a number of subsequent studies, that were reviewed in 2008 by Berto et al. [26] In particular, a study performed in patients with AR from Czech Republic compared directly the treatment with the two forms of AIT (SCIT and SLIT) and only drug treatment for 3 years. The mean direct cost per patient was estimated in €482 for SCIT and €416 for SLIT, a SLIT-treated patient paying more than a SCIT-treated patient for allergen extracts (€72 vs. €55) but paying less for out-of-pocket costs (€176 vs. €255). The figure of direct and indirect costs over the 3-year treatment was €1004 for SCIT and €684 for SLIT [27].

### Studies on the 5-grass pollen tablets

The 5-grass pollen tablets, that contain pollen extracts from Pooideae family (*Anthoxanthum odoratum, Poa pratensis, Dactylis glomerata, Phleum pratense,* and *Lolium perenne*) were approved and registered, based on regulatory large trials that fulfilled all requirements by the European Medicine Agency (EMA) in Europe and Food and Drug Administration (FDA) in the US [28–30]. The 5-grass pollens tablets were accepted for full reimbursement by the Agenzia Italiana del Farmaco (AIFA) [31], with the indication for AR and/or conjunctivitis treatment in adult or pediatric patients (over 5 years) with severe symptoms, by a pre-co-seasonal course of administration. The first study on the cost-effectiveness of 5-grass tablets was conducted by Ruggeri et al., based on post hoc analysis of the VO34.04 and VO53.06 trials [28, 29]. The economic data from the perspective of Italian third-party payer, as well as a societal perspective based on the costs related to the losses of productivity were analyzed [32]. Medication effectiveness was assessed using as main outcome parameter the Quality Adjusted Life Years (QALYs), that is a multi-attribute scale generating a single numeric index of health-related Quality of life (Qol) of patients ranging from 0 (death) to 1 (perfect health). A decision tree modeling the likely outcomes and costs for adults and children with a low, medium, and high score of allergic symptoms was used. Compared to placebo, the 5-grass tablet treatment resulted in 0.127 QALYs in patients with moderate allergic symptoms and in 0.143 QALYs in patients with severe symptoms. The 5-grass pollen tablet treatment had a cost of €1024/QALY for patients with moderate symptoms and €1035/QALY for those with severe symptoms. The authors concluded that, based on the cost-effectiveness for adult patients with moderate to severe AR, the 5-grass tablet should be carefully considered when choosing the management strategy for AR [32]. An investigation conducted in Germany on the outcomes, costs and cost-effectiveness compared the 5-grass tablets to the one-grass tablet and the one-grass extract for subcutaneous injection, using as control the drug treatment alone for grass pollen-induced AR. A Markov model was used to assess the costs and outcomes of a 3-year treatment for a period of 9 years, estimating the treatment efficacy by an indirect comparison of published clinical trials on grass pollen immunotherapy with placebo. The analysis included both public and private health insurance payments. Outcomes were reported as QALYs and symptom-free days. The 5-grass tablet had a predicted cost-utility ratio vs. drug treatment of €14,728 per QALY, with incremental costs corresponding to €1356 and incremental QALYs to 0.092. SLIT with the 5-grass tablet was the prevailing strategy compared to one-grass tablet and SCIT, with incremental costs estimated in −€1142 and −€54 and incremental QALYs estimated in 0.015 and 0.027, respectively. Even though the indirect comparison involving several steps to assess the treatment effects was a limitation, the study suggested that the 5-grass tablet was cost-effective compared to one-grass tablet and injective immunotherapy [33]. In a recent study, the same authors compared, by reviewing the literature and performing meta-analysis and cost-effectiveness analysis the effects and costs of the 5-grass tablet vs. a mix of allergoids for SCIT in grass pollen allergic rhinoconjunctivitis. As for the previous study, a Markov model with a 9 year time length was used to assess the costs and effects of a 3-year-long treatment. Drug acquisition and medical costs, estimates for use of the resources, persistence of AIT and asthma occurrence were obtained from published sources. The analysis was performed from the payer’s perspective in Germany, that includes payments of NHS and additional payments by insurants. A cost-utility ratio of the 5-grass tablet vs. the mix of injectable allergoids of €12,593 per QALY was observed, with predicted incremental costs and QALYs corresponding to €458 and 0.036, respectively. The probability of the 5-grass tablet to be the most cost-effective treatment option was estimated in 76% at a willingness-to-pay threshold of €20,000. These data confirmed the cost-effectiveness of the 5-grass tablet also over SCIT with a mix of allergoids [34].

## Conclusions

In times when a rigid control of expenditures for NHSs is needed, the cost-effectiveness of medical treatments is of paramount importance. Among treatment options for AR, AIT (that includes SCIT and SLIT) has exclusive features, that include the ability to alter the natural history of allergy and to extend its effectiveness, differently from drug treatment, to several years after discontinuing the therapy. A growing bulk of data indicates that SCIT and SLIT may be very advantageous to the healthcare systems [35–38]. However, the pharmacoeconomic advantage demonstrated in optimally performed studies needs to be confirmed by real-life experiences. In fact, in the latest years data showing a poor adherence to long-term SLIT, with a minority of patients completing the recommended 3 years of treatment duration, [39] makes unlikely that the cost effectiveness is achieved when SLIT is abandoned before reaching a duration able to extend the benefit on symptoms and use of drugs over time. This issue needs to be considered by all specialists concerned in this treatment.

## Abbreviations

AR: allergic rhinitis
AIT: allergen immunotherapy
SCIT: subcutaneous immunotherapy
SLIT: sublingual immunotherapy
EMA: European Medicine Agency
FDA: Food and Drug Administration
AIFA: Agenzia Italiana del Farmaco
QALYs: Quality Adjusted Life Years
QoL: quality of life
AS: allergic symptoms
NHS: National Health System
